# One- and two-stage surgical revision of peri-prosthetic joint infection of the hip: a pooled individual participant data analysis of 44 cohort studies

**DOI:** 10.1007/s10654-018-0377-9

**Published:** 2018-04-05

**Authors:** Setor K. Kunutsor, Michael R. Whitehouse, Ashley W. Blom, Tim Board, Peter Kay, B. Mike Wroblewski, Valérie Zeller, Szu-Yuan Chen, Pang-Hsin Hsieh, Bassam A. Masri, Amir Herman, Jean-Yves Jenny, Ran Schwarzkopf, John-Paul Whittaker, Ben Burston, Ronald Huang, Camilo Restrepo, Javad Parvizi, Sergio Rudelli, Emerson Honda, David E. Uip, Guillem Bori, Ernesto Muñoz-Mahamud, Elizabeth Darley, Alba Ribera, Elena Cañas, Javier Cabo, José Cordero-Ampuero, Maria Luisa Sorlí Redó, Simon Strange, Erik Lenguerrand, Rachael Gooberman-Hill, Jason Webb, Alasdair MacGowan, Paul Dieppe, Matthew Wilson, Andrew D. Beswick, The Global Infection Orthopaedic Management Collaboration

**Affiliations:** 1 Musculoskeletal Research Unit, Translational Health Sciences, Bristol Medical School, Southmead Hospital, University of Bristol, Southmead Road, Bristol, BS10 5NB UK; 2 National Institute for Health Research Bristol Biomedical Research Centre, University of Bristol, Bristol, UK; 3 Wrightington, Wigan and Leigh NHS Foundation Trust, Appley Bridge, Wigan, Lancashire WN6 9EP UK; 4 Centre de Référence des Infections Ostéo-Articulaires Complexes, Groupe Hospitalier Diaconesses-Croix Saint-Simon, 125, rue d’Avron, 75020 Paris, France; 5 Department of Orthopaedic Surgery, Chang Gung Memorial Hospital, Chang Gung University College of Medicine, 5 Fu-Hsing Street, Kweishan, 333 Taoyuan Taiwan; 6 Department of Orthopaedics, University of British Columbia, 2775 Laurel St, Vancouver, V5Z 1M9 BC Canada; 7 Centre for Orthopaedic and Hand Surgery, University Hospital, Strasbourg, 10 Avenue Baumann, 67400 Illkirch, France; 8 Division of Adult Reconstruction, Department of Orthopedics, NYU Langone Medical Center, Hospital for Joint Diseases, New York, NY USA; 9 Robert Jones and Agnes Hunt Orthopaedic Hospital NHS Trust, Oswestry, SY10 7AG UK; 10 The Rothman Institute of Orthopaedics, Thomas Jefferson University Hospital, 925 Chestnut Street, 5th Floor, Philadelphia, PA 19107 USA; 11 Department of Orthopaedic Surgery, Santa Casa Medical School, São Paulo, Brazil; 12 Institute of Education and Research of Sírio Libanês Hospital, São Paulo, Brazil; 13 Bone and Joint Infectious Diseases Unit, Department of Orthopaedic and Trauma Surgery, IDIBAPS, Hospital Clinic of Barcelona, University of Barcelona, C/Villarroel 170, 08036 Barcelona, Spain; 14 Severn Pathology Infection Sciences, Pathology Sciences Building, North Bristol NHS Trust, Southmead Hospital, Westbury-on-Trym, Bristol, BS10 5ND UK; 15 Department of Infectious Diseases, IDIBELL, Hospital Universitari de Bellvitge, Feixa Llarga s/n., L’Hospitalet de Llobregat, 08907 Barcelona, Spain; 16 Department of Orthopaedic Surgery, IDIBELL, Hospital Universitari de Bellvitge, Feixa Llarga s/n., L’Hospitalet de Llobregat, 08907 Barcelona, Spain; 17 Cirugía Ortopédica y Traumatología, Hospital Universitario La Princesa, Océano Antártico 41, Tres Cantos, 28760 Madrid, Spain; 18 Department of Infectious Diseases, Parc de Salut Mar, Passeig Marítim 25-29, E-08003 Barcelona, Spain; 19 North Bristol NHS Trust, Southmead Hospital, Bristol, BS10 5NB UK; 20 Medical School, University of Exeter, Exeter, EX1 2LU UK; 21 Princess Elizabeth Orthopaedic Centre, Royal Devon and Exeter NHS Foundation Trust, Barrack Road, Exeter, EX2 5DW UK

**Keywords:** Prosthesis related infection, Total hip replacement, Reoperation, Revision, Re-infection, One-stage, Two-stage, Meta-analysis

## Abstract

One-stage and two-stage revision strategies are the two main options for treating established chronic peri-prosthetic joint infection (PJI) of the hip; however, there is uncertainty regarding which is the best treatment option. We aimed to compare the risk of re-infection between the two revision strategies using pooled individual participant data (IPD). Observational cohort studies with PJI of the hip treated exclusively by one- or two-stage revision and reporting re-infection outcomes were retrieved by searching MEDLINE, EMBASE, Web of Science, The Cochrane Library, and the WHO International Clinical Trials Registry Platform; as well as email contact with investigators. We analysed IPD of 1856 participants with PJI of the hip from 44 cohorts across four continents. The primary outcome was re-infection (recurrence of infection by the same organism(s) and/or re-infection with a new organism(s)). Hazard ratios (HRs) for re-infection were calculated using Cox proportional frailty hazards models. After a median follow-up of 3.7 years, 222 re-infections were recorded. Re-infection rates per 1000 person-years of follow-up were 16.8 (95% CI 13.6–20.7) and 32.3 (95% CI 27.3–38.3) for one-stage and two-stage strategies respectively. The age- and sex-adjusted HR of re-infection for two-stage revision was 1.70 (0.58–5.00) when compared with one-stage revision. The association remained consistently absent after further adjustment for potential confounders. The HRs did not vary importantly in clinically relevant subgroups. Analysis of pooled individual patient data suggest that a one-stage revision strategy may be as effective as a two-stage revision strategy in treating PJI of the hip.

**Electronic supplementary material:**

The online version of this article (10.1007/s10654-018-0377-9) contains supplementary material, which is available to authorized users.

## Introduction

Hip replacement is one of the most common surgical procedures. In the UK, over 95,000 primary procedures were performed in 2015 [1, 2]. In 2010, it was estimated that 2.5 million Americans were living with a hip replacement [3]. Peri-prosthetic joint infection (PJI) is a serious adverse event affecting approximately one percent of patients with a primary hip joint replacement [4] PJI has a major negative effect on patients’ quality of life [5–7], and to avoid the need for excision arthroplasty or amputation, patients and their treating surgeons face complex and protracted treatments.

In 1985, Fitzgerald and Jones described a series of two-stage revisions for the treatment of infected hip implants [8]. With this two-stage strategy, the artificial hip joint is removed and replacement delayed for several months until clear evidence of infection eradication is obtained. An alternative one-stage revision procedure was in use from 1976 at the Endo-Klinik in Hamburg with the implant removed and replaced in one operation [9]; however the two-stage strategy has traditionally been considered the gold standard for PJI treatment [10].

Given the absence of a robust randomised controlled trial (RCT), the effectiveness of the two strategies have been compared using aggregate data from case series [11–13]. In the most recent review of 98 studies, we reported 2-year re-infection rates of about 8% following both one- or two-stage surgical revision for PJI of the hip [14]. Our findings also showed that re-infection outcomes were generally consistent for the revision strategies across important patient characteristics and surgical factors. Some features of our review limited the generalisability of the findings. First, a detailed assessment of the definition of re-infection could not be undertaken as this was not clearly reported in the majority of studies. Second, our aim was to include studies with at least 2 years of follow-up following revision surgery, but this information was not always available.

In the absence of robust evidence from a carefully designed RCT, access to individual level data from published studies could address the existing uncertainties and enable: (1) a consistent approach to the definition of outcomes; (2) a common approach across studies to statistical analyses; and (3) improved generalisability through inclusion of patients from key prospective studies worldwide.

In this context, we aimed to: (1) compare baseline and clinical characteristics of patients undergoing one-stage and two-stage revision surgery following PJI of the hip; (2) compare the risk of re-infection between the two strategies; and (3) examine the risk of re-infection according to a range of clinically relevant characteristics. To achieve our aims, we established the Global Infection Orthopaedic Management (INFORM) collaboration. This international consortium has allowed central collation and harmonisation of individual participant data (IPD) on 1856 patients from 44 cohorts based in 13 different countries across 4 continents.

## Methods

### Data sources and search strategy

We conducted this systematic review and IPD pooled analysis using a predefined protocol registered in the PROSPERO International prospective register of systematic reviews (CRD42015016664) [15], and in accordance with methods recommended by the IPD Meta-analysis Methods Group of the Cochrane Collaboration [16], guidance of Riley and colleagues [17], and the Preferred Reporting Items for Systematic Reviews and Meta-Analyses of Individual Participants Data (PRISMA-IPD) guidelines [18] (see Appendix Supplement 1). We sought IPD from studies identified through systematic searches of MEDLINE, EMBASE, Web of Science, Cochrane Database of Systematic Reviews, Cochrane Central Register of Controlled Trials, and the WHO International Clinical Trials Registry Platform from March 2011 (date of our search for the previous review [13]) to February 2015 and subsequently updated to August 2016. The computer-based searches combined free text and medical subject headings and combination of key words related to hip replacement, infection, and revision with focus on one- and two-stage surgeries. There were no restrictions on language. Studies were also identified from reference lists of all retrieved articles and other relevant publications, including reviews and meta-analyses, and discussions with investigators of unpublished studies. Further details on the search strategy are presented in Appendix Supplement 2. No separate ethical approval was required for the conduct of this study, as any necessary ethical approval was obtained for each of the individual studies contributing data to this pooled analysis.

### Eligibility criteria

Cohort studies were eligible if they met the following inclusion criteria: (1) generally unselected patients with PJI of the hip (i.e., patients’ representative of the general patient population); (2) patients treated exclusively by one-stage or two-stage revision; (3) and patients with at least 2 years of follow-up for re-infection outcomes. Peri-prosthetic joint infection was mainly diagnosed on the basis of both the presence of clinical symptoms and the results of microbiological culture from joint aspiration before surgery and/or during surgery. Majority of studies defined PJI based on diagnostic criteria proposed by the Musculoskeletal Infection Society: positive joint fluid cultures, joint fluid cell count and differentials, inflammatory markers [C-reactive protein (CRP) and erythrocyte sedimentation rate (ESR), presence of a sinus tract, gross purulence observed at the time of surgery, and a positive histological exam for acute inflammation in tissues obtained during surgery. Studies that reported case series of methods in selected groups of patients (such as subsamples of patients who received revision in one- or two-stages or patients with a specific infection such as fungal infections) were excluded from the review.

### Global Infection Orthopaedic Management (INFORM) collaboration

Details of the establishment of the Global INFORM collaboration have been reported previously in the published protocol [15]. Briefly, investigators of eligible studies identified by the literature search strategy and well-known investigators in the field, were contacted by email or letter, provided with a summary of the study protocol, and invited to join the collaboration if they had the relevant data available. Investigators expressing interest to collaborate in this effort were then provided with full details of the study protocol.

### Data collection

Investigators were provided with a list of relevant study variables that could be used in the analyses (Appendix Supplement 3). Data from each study were obtained using a standardised spreadsheet, and data dictionaries were also requested. Details of contributing cohorts are presented in Appendix Supplement 4. The raw data were examined and inconsistencies or irregularities were clarified with the investigators. Individual level data collected was coded and entered into a single database. Additional studies were included where useable data was tabulated in published articles.

### Outcome

The primary outcome variable was clinically diagnosed re-infection, i.e. recurrence of infection by the same organism(s) and/or re-infection with a new organism(s). Patients contributed only the first re-infection recorded after revision during follow-up. Outcomes were censored if a patient was lost to follow-up.

### Statistical analysis

Descriptive statistics were used to summarise baseline characteristics according to the type of revision strategy. We reported mean, standard deviation (SD), median, and interquartile range (IQR) for continuous variables, and proportions for categorical variables. The risks of re-infection recorded during follow-up comparing the two-stage with the one-stage (reference category) strategy were assessed using Cox proportional shared frailty models [19], after confirmation of no major departure from the proportionality of hazards assumptions [20]. Because the treatment variable (i.e. revision strategy) only varied between studies/cohorts, inferences could only be made based on differences in re-infection rates between studies using either treatment strategy. A stratified Cox model was therefore not suitable in this scenario as the “treatment strategy” did not vary within studies. We employed a shared frailty model, which is an extension of the Cox proportional hazards model and provides a suitable way to introduce random effects in the model to account for unobserved heterogeneity. The random effect (the frailty) has a multiplicative effect on the hazard function of a cluster of individuals (cohort in this case). For each model, we included a frailty term at the cohort level to allow for dependence of individuals within each cohort. Survival curves comparing the one- and two-stage strategies were calculated using unadjusted Kaplan–Meier estimates and compared using the log-rank test. Hazard ratios (HRs) with 95% confidence intervals (CIs) were calculated with progressive adjustment for age, sex, comorbidities (Charlson comorbidity index [21]), previous hip surgery, and type of infecting organism (“difficult to treat versus “not difficult to treat” [22, 23]; Appendix Supplement 5). These covariates were selected on the basis of their role as potential confounders and evidence from previous research. Subgroup analyses were conducted using interaction tests to assess statistical evidence of any differences in HRs across categories of pre-specified individual level characteristics, specifically: sex, age group, previous hip surgery, and type of infecting organism. A two-sided *P* value less than 0.05 was considered statistically significant throughout and all analyses were conducted using Stata version 14 (StataCorp, College Station, Texas, USA).

## Results

### Study identification and selection

**Fig. 1:**
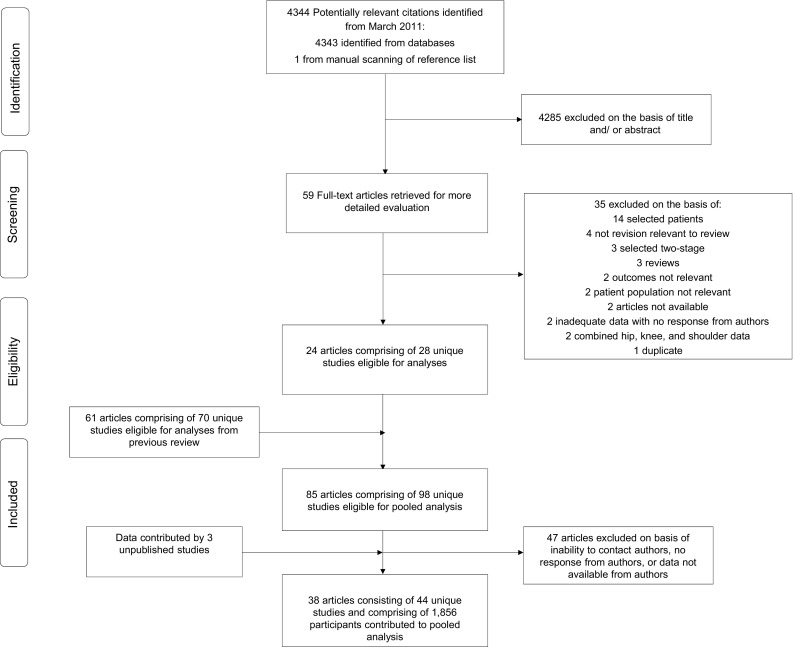
Selection of studies included in the individual pooled data analysis

Figure 1 shows the inclusion and exclusion of studies. Our systematic literature search identified 4344 potentially relevant citations. After screening titles and abstracts, 59 articles remained for further evaluation. Following detailed assessments, 35 articles were excluded. The remaining 24 articles (based on 28 unique studies) and 61 articles (based on 70 unique studies) identified from our previous review [13], were potentially eligible for the pooled analysis. Of this number and in addition to three studies based on unpublished data, we had access to individual level data from 44 cohort studies. Overall, there were 13 one-stage and 31 two-stage studies based in 13 countries (from North and South America, Europe, and Asia) (Appendix Supplements 4 and 6).

### Baseline and follow-up characteristics

**Fig. 2:**
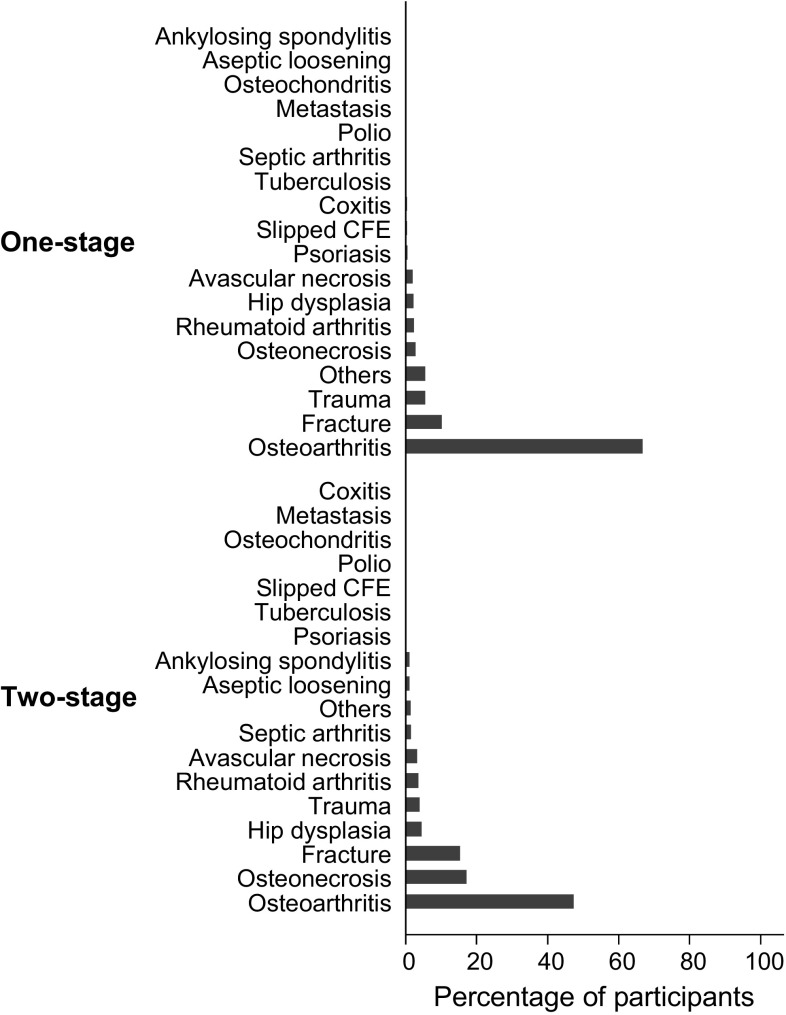
Indications for index implantation by type of revision strategy

**Fig. 3:**
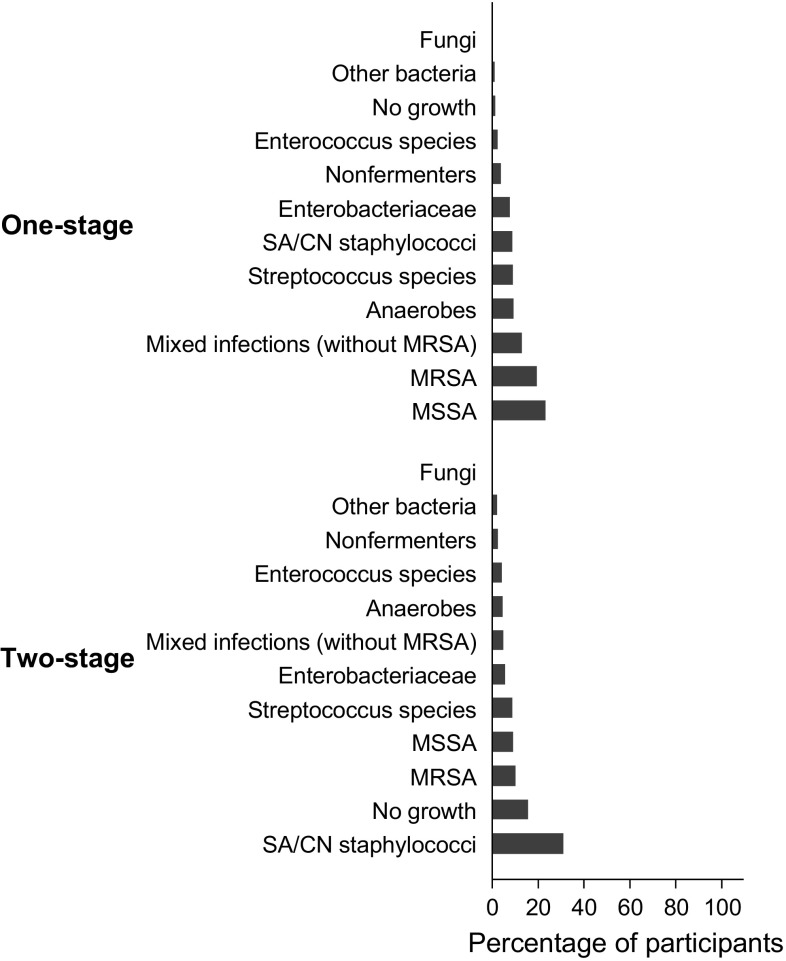
Type of infecting microorganism after index implantation by type of revision strategy

Summary baseline and follow-up characteristics of the 1856 patients with PJI of the hip treated by one- or two-stage revision that contributed to the analyses are shown in Table 1. The mean (SD) age of overall participants at baseline was 65 (13) years and 53% were men. A total of 884 patients received one-stage revision and 972 patients received two-stage revision. The median (interquartile range) follow up time was 4.2 (2.0–8.1) years in the one-stage group and 3.3 (2.0–5.9) years in the two-stage group. During follow-up, 88 (10.0%) participants experienced a re-infection in the one-stage group compared with 134 (13.8%) in the two-stage group. Although the proportion of men, mean BMI, proportion of patients having a previous procedure to treat infection, and median baseline Harris Hip Score (HHS) between the two treatment groups were generally similar, several baseline characteristics and follow-up data were not balanced between one- and two-stage groups. The one-stage revision group had older patients on average and had a higher proportion of patients with previous PJI and previous hip surgery (other than the index surgery) compared with their two-stage counterparts. In addition, the one-stage revision group had higher median levels of baseline blood circulating CRP and a higher proportion of patients presenting with an abscess, sinus, draining wound, or fistula before revision. In the two-stage group, a higher proportion of patients had a history of diabetes and other comorbidities compared with one-stage patients. The most common indication for the index implantation for both groups was osteoarthritis. This was followed by fractures in the one-stage group and osteonecrosis in the two-stage group (Fig. 2). The most common cultured microorganism responsible for a PJI after the index operation in the one-stage group was methicillin-sensitive *Staphylococcus* (*S.*) *aureus* (MSSA); whereas it was *S. aureus* or coagulase-negative staphylococci (CoNS) in the two-stage group. Compared to the one-stage group, there was a large percentage of negative cultures in the two-stage group (Fig. 3). The median times to onset of infection from index implantation and from infection to revision surgery were longer in one-stage revision strategy patients compared with two-stage patients. The median duration of antibiotic use after revision was considerable longer in the one-stage group compared with the two-stage group. However, the median duration of antibiotic therapy between stages for the two-stage revision group was about twice as long as after revision surgery in the one-stage group. Thus, patients treated with two-stage revision received a longer duration of antibiotics over the entire course of treatment (median, 18.3 weeks) compared with those treated with one-stage (median, 12.6 weeks).

**Table 1:** Summary of baseline characteristics and follow-up data in patients undergoing one- or two-stage revision

	Overall	One-stage revision	Two-stage revision	*P* value
Total number of participants	1856	884	972	
Socio-demographic characteristics				
Gender	N = 1743	N = 864	N = 879	0.922
Males, n (%)	926 (53.1)	458 (53.0)	468 (53.2)	
Females, n (%)	817 (46.9)	406 (47.0)	411 (46.8)	
Age at baseline (years), mean (SD)	65.1 (13.0)	66.8 (12.4)	63.4 (13.3)	< 0.001
Smoking	N = 365	N = 56	N = 309	0.151
Yes, n (%)	86 (23.6)	9 (16.1)	77 (24.9)	
No, n (%)	279 (76.4)	47 (83.9)	232 (75.1)	
History of high alcohol consumption	N = 110	N = 0	N = 110	
Yes, n (%)	6 (5.5)		6 (5.5)	
No, n (%)	104 (94.6)		104 (94.6)	
Physical measurements				
	N = 631	N = 269	N = 362	
Body mass index in kg/m^2^, mean (SD)	27.6 (6.6)	27.5 (5.9)	27.8 (7.0)	0.580
Medical and surgical history				
History of diabetes	N = 803	N = 282	N = 521	0.028
Yes, n (%)	131 (16.3)	35 (12.4)	96 (18.4)	
No, n (%)	676 (83.7)	247 (87.6)	425 (81.6)	
History of hypertension	N = 340	N = 157	N = 183	0.501
Yes, n (%)	119 (35.0)	52 (33.1)	67 (36.6)	
No, n (%)	221 (65.0)	105 (66.9)	116 (63.4)	
History of CVD	N = 403	N = 161	N = 242	0.714
Yes, n (%)	99 (24.6)	38 (23.6)	61 (25.2)	
No, n (%)	304 (75.4)	123 (76.4)	181 (74.8)	
Comorbidity index	N = 785	N = 282	N = 503	< 0.001
No previously recorded disease categories, n (%)	256 (32.6)	45 (16.0)	211 (42.0)	
One or two disease categories, n (%)	433 (55.2)	212 (75.2)	221 (43.9)	
More than two disease categories, n (%)	96 (12.2)	25 (8.9)	71 (14.1)	
History of previous PJI	N = 321	N = 120	N = 201	< 0.001
Yes, n (%)	62 (19.3)	47 (39.2)	15 (7.5)	
No, n (%)	259 (80.7)	73 (60.8)	186 (92.5)	
Previous hip surgery	N = 1060	N = 809	N = 251	< 0.001
Yes, n (%)	825 (77.8)	748 (92.5)	77 (30.7)	
No, n (%)	235 (22.2)	61 (7.5)	174 (69.3)	
Hip involved in index implantation	N = 1233	N = 632	N = 601	0.863
Right, n (%)	676 (54.8)	348 (55.1)	328 (54.6)	
Left, n (%)	557 (45.2)	284 (44.9)	273 (45.4)	
Characteristics of infection before revision procedure				
Previous procedure performed to treat infection	N = 541	N = 277	N = 264	0.977
Yes, n (%)	137 (25.3)	70 (25.3)	67 (25.4)	
No, n (%)	404 (74.7)	207 (74.7)	197 (74.6)	
Presence of abscess, sinus, draining wound, or fistula at presentation	N = 588	N = 278	N = 310	0.035
Yes, n (%)	160 (27.2)	87 (31.3)	73 (23.6)	
No, n (%)	428 (72.8)	191 (68.7)	237 (76.5)	
Time from index implantation to infection (weeks), median (IQR)	102.7 (36.6–299.2)	154.3 (51.4–350.1)	102.6 (32.6–268.5)	0.142
Time from infection to revision procedure (weeks), median (IQR)	20.6 (8.4–51.4)	30.0 (10.2–94.2)	12.9 (6.4–34.3)	< 0.001
Baseline data before revision				
C-reactive protein (mg/l), [N] median (IQR)	[680] 18.9 (6.1–54.0)	[248] 22.5 (9.0–56.5)	[432] 17.1 (5.8–50.5)	0.052
Erythrocyte sedimentation rate (mm/h), [N] median (IQR)	[371] 47 (26–73)	[70] 41 (28–55)	[301] 51 (25–76)	0.114
Neutrophils/µl, [N] median (IQR)	[69] 4520 (2800–6000)	[23] 4800 (4100–6000)	[46] 3835 (99–5980)	0.044
WBC/µl, [N] median (IQR)	[285] 7380 (6020–9090)	[178] 7100 (5920–8580)	[107] 8030 (6630–10860)	0.002
Harris Hip Score, [N] median (IQR)	[171] 55.0 (48.0–60.0)	[12] 55.5 (43.5–63.5)	[159] 55–0 (48·0–60.0)	0.656
Characteristics of revision procedure and management				
Type of re-implantation	N = 122	N = 89	N = 33	0.201
Cemented, n (%)	91 (74.6)	65 (73.0)	26 (78.8)	
Cementless, n (%)	23 (18.9)	16 (18.0)	7 (21.2)	
Hybrid, n (%)	8 (6.6)	8 (9.0)	0 (0.0)	
Antibiotics in cement	N = 1092	N = 758	N = 334	< 0.001
Yes, n (%)	750 (68.7)	584 (77.0)	166 (49.7)	
No, n (%)	342 (31.3)	174 (23.0)	168 (50.3)	
Nature of spacer used	–	–	N = 293	
Unknown, n (%)	–	–	2 (0.7)	
Articulated, n (%)	–	–	287 (98.0)	
Static, n (%)	–	–	4 (1.4)	
Type of spacer	–		N = 183	
Unknown, n (%)	–	–	1 (0.6)	
Handmade, n (%)	–	–	167 (91.3)	
Commercial, n (%)	–	–	15 (8.2)	
Antibiotics in spacer	–	–	N = 183	
Yes, n (%)	–		180 (98.4)	
No, n (%)	–		3 (1.6)	
Duration between stages (weeks), median (IQR)	–	–	14.5 (11.0–24.0)	
Duration of antibiotics use between stages (weeks), median (IQR)	–	–	24.0 (4.5–24.0)	
After revision (follow-up)				
Duration of antibiotic use after revision surgery (weeks), median (IQR)	12.1 (6.1–12.6)	12.6 (12.0–12.6)	1.3 (0.5–5.5)	< 0.001
Duration of follow-up (years), median (IQR)	3.7 (2.0–6.9)	4.2 (2.0–8.1)	3.3 (2.0–5.9)	< 0.001
Harris Hip Score at follow up, median (IQR)	86.0 (73.0–93.0)	80.0 (52.0–90.0)	87.0 (78.0–95.0)	0.003
Number of re-infections	222	88	134	

*CVD* cardiovascular disease, *IQR* interquartile range, *MR* methicillin resistant, *MS* methicillin sensitive, *PJI* periprosthetic joint infection, *SD* standard deviation, *WBC* white blood cells

### Revision strategy and risk of re-infection

**Fig. 4:**
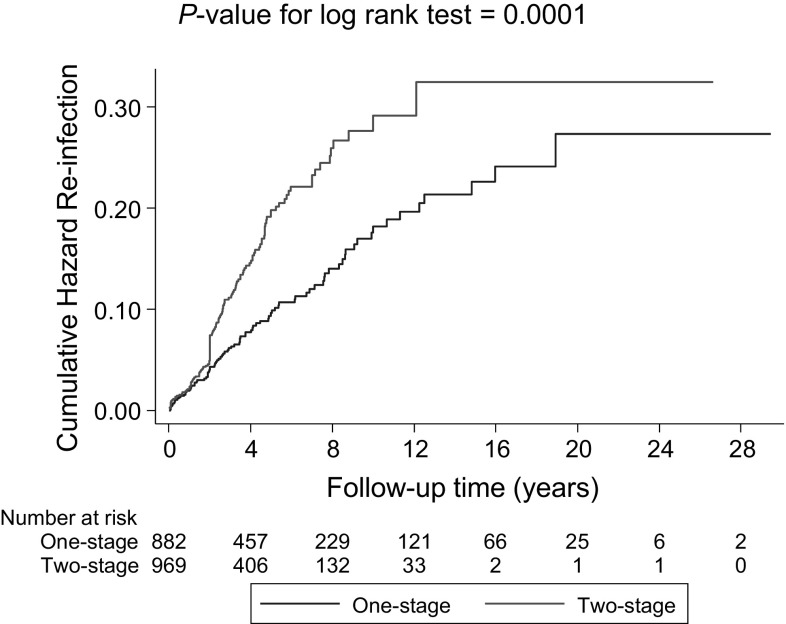
Cumulative hazard curves for re-infection by type of revision strategy

**Fig. 5:**
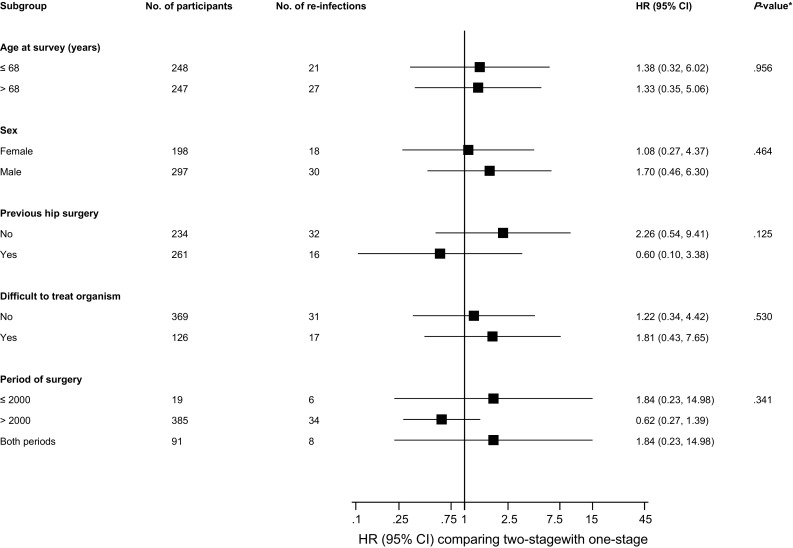
Hazard ratios for re-infection by participant level characteristics. Hazard ratios were adjusted for age, sex, previous hip surgery other than index surgery (yes/no), and difficult to treat organism (yes/no); *CI* confidence interval, *HR* hazard ratio; *P value for interaction. Analysis was limited to 495 participants (comprising 48 re-infections) with available data

During a median (interquartile range) follow-up of 3.7 (2.0–6.9) years, 222 re-infections were recorded. Cumulative hazard curves demonstrated a greater risk of re-infection among two-stage revision strategy participants compared with one-stage revision strategy participants (*P *< 0.001 for log-rank test; Fig. 4). Re-infection rates per 1000 person-years of follow-up across revision strategies were 16.8 (95% CI 13.6–20.7) and 32.3 (95% CI 27.3–38.3) for the one-stage and two-stage strategies respectively. Among 1038 individuals (113 re-infections) with available survival data, comparing two- with one-stage revision, the age-adjusted HR for re-infection was 1.69 (95% CI 0.58–4.98; *P *= 0.338). The corresponding HR remained consistent 1.70 (95% CI 0.58–5.00; *P *= 0.332) on adjusting for sex; and was attenuated to 1.33 (95% CI 0.48–3.69; *P *= 0.583) after further adjustment for previous hip surgery (Table 2). The associations remained absent in analyses restricted to 439 individuals (41 re-infections) with available data on comorbidities and type of infecting organism (Table 2). HRs did not vary importantly by levels or categories of pre-specified patient level characteristics (*P* for interaction > 0.10 for each) (Fig. 5). In a post hoc subgroup analysis by period of surgery, there was no statistically significant evidence of interaction.

**Table 2:** Hazard ratios for re-infection comparing two-stage revision versus one-stage revision adjusted progressively for risk factors

Model	Hazard ratio (95% CI)	*P* value	Hazard ratio (95% CI)	*P* value
	1038 participants (113 re-infections) with available data		439 participants (41 re-infections) with available data	
Model 1	1.69 (0.58–4.98)	0.338	1.65 (0.44–6·20)	0.460
Model 2	1.70 (0.58–5.00)	0.332	1.66 (0.44–6·24)	0.454
Model 3	1.33 (0.48–3.69)	0.583	1.57 (0.45–5·51)	0.484
Model 4	–	–	1.59 (0.39–6·55)	0.520
Model 5	–	–	1.71 (0.39–7·50)	0.479

Model 1: adjusted for age

Model 2: model 1 plus sex

Model 3: model 2 plus previous hip surgery other than index surgery (yes/no)

Model 4: model 3 plus Charlson comorbidity index (no previous disease/one or two disease categories/more than two disease categories)

Model 5: model 4 plus difficult to treat organism (yes/no)

## Comment

### Key findings

This large-scale study involving pooled analysis of individual level data from 44 observational cohort studies was conducted in an attempt to address the uncertainties regarding the effectiveness of one-stage and two-stage revision strategies for treating PJI of the hip, using re-infection as the outcome of interest. With the exception of average BMI, proportions of men and patients having a previous procedure to treat infection, and median baseline HHS, which were similar between the two treatment groups; there were differences in baseline and follow-up characteristics between one- and two-stage revision strategy patients. Males were slightly overrepresented in both treatment groups, a finding which was not unexpected given that male sex is an established risk factor for PJI [24, 25]. The proportions of patients with a previous hip surgery other than the index surgery as well as a previous PJI were higher in the one-stage revision strategy group compared with the two-stage group. Patients in the one-stage revision group seemed to have severe PJI at presentation compared with the two-stage group, given their higher levels of circulating CRP and higher proportion presenting with an abscess, sinus, draining wound, or fistula. These findings were unexpected, as patients with severe PJI often undergo a two-stage revision to facilitate additional antimicrobial strategies. Given the more limited opportunities for antibiotic therapy associated with it, the one-stage revision strategy has been traditionally thought to expose patients to a higher risk of re-infection by residual bacteria [26]; and it has been suggested this strategy should only be used in selected cases, such as patients with known organisms and sensitivities, non-immunocompromised patients, as well as absence of a sinus tract [27, 28]. Our results also showed that MSSA was the most commonly isolated microorganism responsible for a PJI in the one-stage revision group. Compared with one-stage revision patients, the two-stage group had a higher proportion of patients with comorbidities. *Staphylococcus* species were the most common causative organisms for PJI in both treatment groups, results which are consistent with the literature [23, 29, 30]. In addition, a large percentage of two-stage patients had negative cultures compared with the one-stage group, which reflects evidence that the one-stage is commonly used in patients with known organisms and sensitivities [27]. Results on the time to onset of infection from index implantation suggested that a majority of PJIs in the one-stage group were late infections (more than 24 months after surgery), while those of the two-stage group were delayed infections (3–24 months after surgery) [31]. Given that late infections are mostly acquired by haematogenous seeding [23], this might account for the severity of PJI in the one-stage revision group.

Unadjusted cumulative hazard curves suggested a higher re-infection rate for the two-stage revision strategy compared with one-stage revision; however, given the imbalance between several baseline sociodemographic and clinical characteristics, such unadjusted results are likely to be confounded. In multivariate analyses, there was no evidence of a statistically significant increased risk of re-infection, when the two-stage revision strategy was compared with the one-stage revision strategy. However, the findings suggested there might be a higher risk of re-infection in the two-stage revision group compared to the one-stage group. The statistically non-significant associations remained consistent across clinically relevant subgroups. Given that the data collected for the current analysis spanned the period 1971 through 2011 and which might constitute a potential source of confounding for our analyses, we conducted a subgroup analysis by period of surgery and there was no evidence of effect modification by period of surgery. However, there was a suggestion of a protective effect for the period beyond the year 2000; a finding which might reflect the adoption of improved surgical strategies and use of newer and more effective antimicrobial therapies in recent times compared to previous years. However, given the small samples in these subgroups for analysis, further investigation is required.

### Comparison with previous work

We are unable to directly compare the current findings with previous work; because this is to our knowledge, the first pooled analysis of individual level data from observational cohort studies based in different countries that have reported re-infection outcomes following one- or two-stage surgical revision for infected hip prostheses. However, our overall results, which suggest that the one-stage revision strategy may be as effective as the two-stage revision strategy in treating infected hip prostheses, seem to concur and further extend that of previous aggregate reviews conducted on the topic. In a review including 38 one-stage and 60 two-stage revision strategy studies, we demonstrated similar re-infection rates following one- or two-stage surgical revision for infected hip prosthesis [14]. Other similar reviews have also reported findings which suggest no significant superiority of either revision strategy over the other. Leonard and colleagues in a review of nine studies comparing re-infection rates between one- and two-stage revision strategies, reported that one-stage revision was associated with similar re-infection rates when compared with two-stage revision, and had superior functional outcomes [32]. Lange and colleagues in a meta-analysis involving 36 studies, reported results which indicated that there were three additional re-infections per 100 patients with infected hip prosthesis when a one-stage revision was performed compared to a two-stage revision; however, the risk estimates were imprecise with overlapping confidence intervals, demonstrating no clear evidence of a superior revision strategy [33].

### Implications of findings

The current findings, as well as consistent findings from several previous reviews, suggest that the one-stage revision strategy may be as effective as the two-stage strategy in treating many patients with PJI of the hip. These results are very relevant and may have clinical implications for orthopaedic practice. For several decades, the two-stage revision strategy has been presumed to be more effective that the one-stage for treating PJIs [23, 34]. However, in the absence of RCTs, several individual observational cohorts, as well as reviews, have consistently failed to show clear supportive evidence for the two-stage strategy being more effective compared with the one-stage strategy. Our finding of a null association is therefore not unexpected as it confirms speculations that the two revision strategies may have comparable effectiveness for treating PJI of the hip. In unadjusted analyses which employed the entire sample in the dataset, there was statistically significant evidence of an association between the two-stage strategy and higher risk of re-infection. Therefore, it is possible that our null results on multivariate analyses could be attributed to low power, especially given the imprecise estimates (wide confidence intervals). Although claimed to be a more effective revision strategy, the two-stage strategy has several drawbacks. In addition to the significant pain and functional impairment, longer hospitalisation periods, and increased risk of mortality associated with this strategy [12, 34, 35], it is known to be associated with higher healthcare costs compared to one-stage revision [36]. For example, within the UK National Health System (NHS), the cost of surgical revision of an infected hip replacement is estimated to be about £22 000 [37]. A retrospective cost analysis performed in a hospital in France estimated the average cost (excluding social expenses) of a one-stage revision for an infected total hip replacement to be €31 133, with a two-stage procedure costing 1.7 times more than the one-stage alternative [36]. Furthermore, we have shown that patients with two-stage revision also receive a longer duration of antibiotics. There has been an increase in the use of the one-stage revision strategy [38–40] after its introduction several decades ago [9]. Despite the perceived drawback of exposing patients to a higher risk of re-infection by any residual bacteria [26], and the limited opportunities for additional antibiotic therapy; the one-stage strategy has major potential advantages for patients which include the need for fewer surgical procedures, shorter hospitalisation admissions, reduced duration of antibiotic use, less time with functional limitation and uncertainty, as well as economic benefits.

As a result of increasing life expectancy, there is a growing healthcare burden due to osteoarthritis [41], which will result in a projected increase in the numbers of primary hip replacements as well as those requiring revision surgery for PJI of the hip [42, 43]. Indeed, analysis of data for England and Wales using the National Joint Registry suggest that the volume of primary and revision hip replacements will increase by 134 and 31%, respectively between 2012 and 2030 [43]. Compared with primary hip replacement procedures, the cost of revision surgery is higher; with revision for PJI being more expensive than aseptic revisions [37]. Given the high financial costs and increased burden on resources associated especially with the two-stage revision strategy, there is a need for optimisation of resources within the current economic climate. The evidence suggests that the two revision strategies have comparable effectiveness in the control of infection in patients with peri-prosthetic hip infection. Our findings also show that the one-stage strategy is an appropriate treatment strategy for patients with characteristics that had previously been thought to be inappropriate for one-stage revision, such as those with sinus tracts at time of presentation. The overall findings suggest that the one-stage strategy might be a preferable strategy for orthopaedic surgeons performing revision surgeries for PJI of the hip.

### Strengths and limitations of the study

Several strengths of this study merit consideration. We have conducted the first pooled analysis of individual level data from observational cohort studies comparing re-infection rates among patients with PJI of the hip who have undergone one- or two stage revision. Although previous aggregate reviews conducted on the topic have included a larger number of studies, the current analysis is unique in the following ways: (1) compared with single-country studies, our study pooled individual level data contributed by study investigators across four continents which enhanced generalisability of the findings; (2) there was a more consistent approach to the definition of re-infection outcomes; (3) it ensured that participants with at least 2 years of follow-up were included in the analyses; (4) there was a common approach across studies to statistical analyses; and (5) analyses included adjustment for relevant confounders which enabled reliable assessment of the treatment effects, given the biases associated with unadjusted results. Despite the novelty and strengths of the current study, there are several limitations which deserve consideration. A main limitation was that because the revision strategy only varied between cohorts, a head-to-head comparison of the two revision strategies could not be made and appropriate inferences could only be made based on differences in re-infection rates between studies using either treatment strategy. However, given the clustered nature of the survival data, we employed a shared frailty Cox proportional model to account for any unobserved heterogeneity. The majority of studies were unable to contribute all relevant clinical data, which precluded adjustment for a comprehensive panel of potential confounders, thereby introducing the possibility of residual confounding. Given the sparcity of the data contributed by different studies, multiple imputation was not considered; as it is a challenging process in such situations and is known to produce inaccurate imputations of the missing values or does not appear to preserve relationships among variables [44]. We were also unable to conduct detailed analyses by clinically relevant subgroups such as type of PJI (early vs delayed vs late), BMI, duration of antibiotic therapy, and by population (geographical region) because of the lack of data. Apart from the control of infection, maintenance of joint function is also considered as an important factor for a successful outcome following one- or two stage revision [45, 46]. We were unable to compare the two revision strategies using measures of joint function such as the Western Ontario & McMaster Universities Arthritis Index (WOMAC) Index, a validated patient-reported outcome measure of hip pain, function and stiffness widely used in joint replacement research [47]. A number of qualitative studies (including one by our group) focusing on outcomes after joint surgery, have shown that patients are more concerned with pain and joint function (patient-centred outcome measures) rather than clinical indices such as re-infection rates [5, 48]. Because we included populations representative of patients in general clinical practice, the results cannot be generalised to selected patient populations such as immunocompromised patients, culture negative patients, and those with periprosthetic fungal infections. The findings should therefore be interpreted in context of the limitations available. Ideally, to compare the effectiveness of these two revision strategies will require evidence from a carefully designed RCT. However, given the low incidence of PJI after total hip replacement, an appropriate definitive RCT with re-infection as the primary outcome may be unlikely in the short term. Lange and colleagues report that a sample size of more than 3500 infected patients would be needed to investigate the superiority of the two-stage over one-stage revision with statistical precision, using re-infection as an outcome [33]. Within our INFection ORthopaedic Management (INFORM) Programme, which is involved in developing and establishing optimum management strategies for PJIs, there is an ongoing trial to determine whether there is a difference in patient-reported outcome measures (primary outcome) between one-stage and two-stage revision surgeries for patients with PJI of the hip (INFORM; Current controlled trials ISRCTN10956306) [49]. Results from this study may help to elucidate and address differences in the effectiveness of these two revision strategies.

In conclusion, analysis of pooled individual patient data suggests that a one-stage revision strategy may be as effective as a two-stage revision strategy in treating PJI of the hip.

## Electronic supplementary material

Below is the link to the electronic supplementary material.

Supplementary material 1 (DOC 176 kb)
