# Supplementary material for: One- and two-stage surgical revision of peri-prosthetic joint infection of the hip: a pooled individual participant data analysis of 44 cohort studies
Source: Eur J Epidemiol. 2018 Apr 5;33(10):933–46. doi: 10.1007/s10654-018-0377-9 (PMC6153557; doi:10.1007/s10654-018-0377-9)
Supplement: Supplementary file 1 — Supplementary material 1 (DOC 176 kb) [file 10654_2018_377_MOESM1_ESM.doc]

**SUPPLEMENTARY MATERIAL**

**One- and two-stage surgical revision of peri-prosthetic joint infection of the hip: A pooled individual participant data analysis of 44 cohort studies**

| **Appendix Supplement 1** | PRISMA checklist |
| --- | --- |
| **Appendix Supplement 2** | Literature search strategy |
| **Appendix Supplement 3** | Study variables requested from investigators |
| **Appendix Supplement 4** | Characteristics of studies contributing data to the pooled analysis |
| **Appendix Supplement 5** | Classification of infecting organisms based on difficulty in treating |
| **Appendix Supplement 6** | Main references of studies contributing data to the pooled analysis |

**Appendix Supplement 1.** PRISMA-IPD Checklist

| **PRISMA-IPD**  **Section/topic** | **Item No** | **Checklist item** | **Reported on page** |
| --- | --- | --- | --- |
| **Title** | | | |
| Title | 1 | Identify the report as a systematic review and meta-analysis of individual participant data. | 1 |
| **Abstract** | | | |
| Structured summary | 2 | Provide a structured summary including as applicable: | 3 |
|  |  | **Background**: state research question and main objectives, with information on participants, interventions, comparators and outcomes. |  |
|  |  | **Methods**: report eligibility criteria; data sources including dates of last bibliographic search or elicitation, noting that IPD were sought; methods of assessing risk of bias. |  |
|  |  | **Results**: provide number and type of studies and participants identified and number (%) obtained; summary effect estimates for main outcomes (benefits and harms) with confidence intervals and measures of statistical heterogeneity. Describe the direction and size of summary effects in terms meaningful to those who would put findings into practice. |  |
|  |  | **Discussion:** state main strengths and limitations of the evidence, general interpretation of the results and any important implications. |  |
|  |  | **Other:** report primary funding source, registration number and registry name for the systematic review and IPD meta-analysis. |  |
| **Introduction** | | | |
| Rationale | 3 | Describe the rationale for the review in the context of what is already known. | 4-5 |
| Objectives | 4 | Provide an explicit statement of the questions being addressed with reference, as applicable, to participants, interventions, comparisons, outcomes and study design (PICOS). Include any hypotheses that relate to particular types of participant-level subgroups. | 5 |
| **Methods** | | | |
| Protocol and registration | 5 | Indicate if a protocol exists and where it can be accessed. If available, provide registration information including registration number and registry name. Provide publication details, if applicable. | 5 |
| Eligibility criteria | 6 | Specify inclusion and exclusion criteria including those relating to participants, interventions, comparisons, outcomes, study design and characteristics (e.g. years when conducted, required minimum follow-up). Note whether these were applied at the study or individual level i.e. whether eligible participants were included (and ineligible participants excluded) from a study that included a wider population than specified by the review inclusion criteria. The rationale for criteria should be stated. | 6 |
| Identifying studies - information sources | 7 | Describe all methods of identifying published and unpublished studies including, as applicable: which bibliographic databases were searched with dates of coverage; details of any hand searching including of conference proceedings; use of study registers and agency or company databases; contact with the original research team and experts in the field; open adverts and surveys. Give the date of last search or elicitation. | 6 |
| Identifying studies - search | 8 | Present the full electronic search strategy for at least one database, including any limits used, such that it could be repeated. | Appendix Supplement 2 |
| Study selection processes | 9 | State the process for determining which studies were eligible for inclusion. | 6 |
| Data collection processes | 10 | Describe how IPD were requested, collected and managed, including any processes for querying and confirming data with investigators. If IPD were not sought from any eligible study, the reason for this should be stated (for each such study). | 6 |
|  |  | If applicable, describe how any studies for which IPD were not available were dealt with. This should include whether, how and what aggregate data were sought or extracted from study reports and publications (such as extracting data independently in duplicate) and any processes for obtaining and confirming these data with investigators. |  |
| Data items | 11 | Describe how the information and variables to be collected were chosen. List and define all study level and participant level data that were sought, including baseline and follow-up information. If applicable, describe methods of standardising or translating variables within the IPD datasets to ensure common scales or measurements across studies. | Appendix Supplement 3 |
| IPD integrity | A1 | Describe what aspects of IPD were subject to data checking (such as sequence generation, data consistency and completeness, baseline imbalance) and how this was done. | 6 |
| Risk of bias assessment in individual studies. | 12 | Describe methods used to assess risk of bias in the individual studies and whether this was applied separately for each outcome. If applicable, describe how findings of IPD checking were used to inform the assessment. Report if and how risk of bias assessment was used in any data synthesis. | 7 |
| Specification of outcomes and effect measures | 13 | State all treatment comparisons of interests. State all outcomes addressed and define them in detail. State whether they were pre-specified for the review and, if applicable, whether they were primary/main or secondary/additional outcomes. Give the principal measures of effect (such as risk ratio, hazard ratio, difference in means) used for each outcome. | 6-7 |
| Synthesis methods | 14 | Describe the meta-analysis methods used to synthesise IPD. Specify any statistical methods and models used. Issues should include (but are not restricted to):   - Use of a one-stage or two-stage approach. - How effect estimates were generated separately within each study and combined across studies (where applicable). - Specification of one-stage models (where applicable) including how clustering of patients within studies was accounted for. - Use of fixed or random effects models and any other model assumptions, such as proportional hazards. - How (summary) survival curves were generated (where applicable). - Methods for quantifying statistical heterogeneity (such as I^2^ and τ^2^). - How studies providing IPD and not providing IPD were analysed together (where applicable). - How missing data within the IPD were dealt with (where applicable). | **7** |
| Exploration of variation in effects | A2 | If applicable, describe any methods used to explore variation in effects by study or participant level characteristics (such as estimation of interactions between effect and covariates). State all participant-level characteristics that were analysed as potential effect modifiers, and whether these were pre-specified. | 7 |
| Risk of bias across studies | 15 | Specify any assessment of risk of bias relating to the accumulated body of evidence, including any pertaining to not obtaining IPD for particular studies, outcomes or other variables. | 7 |
| Additional analyses | 16 | Describe methods of any additional analyses, including sensitivity analyses. State which of these were pre-specified. | 7 |
| **Results** | | | |
| Study selection and IPD obtained | 17 | Give numbers of studies screened, assessed for eligibility, and included in the systematic review with reasons for exclusions at each stage. Indicate the number of studies and participants for which IPD were sought and for which IPD were obtained. For those studies where IPD were not available, give the numbers of studies and participants for which aggregate data were available. Report reasons for non-availability of IPD. Include a flow diagram. | 8 |
| Study characteristics | 18 | For each study, present information on key study and participant characteristics (such as description of interventions, numbers of participants, demographic data, unavailability of outcomes, funding source, and if applicable duration of follow-up). Provide (main) citations for each study. Where applicable, also report similar study characteristics for any studies not providing IPD. | Appendix Supplements 4 and 6; Table 1 |
| IPD integrity | A3 | Report any important issues identified in checking IPD or state that there were none. | None |
| Risk of bias within studies | 19 | Present data on risk of bias assessments. If applicable, describe whether data checking led to the up-weighting or down-weighting of these assessments. Consider how any potential bias impacts on the robustness of meta-analysis conclusions. | Not applicable |
| Results of individual studies | 20 | For each comparison and for each main outcome (benefit or harm), for each individual study report the number of eligible participants for which data were obtained and show simple summary data for each intervention group (including, where applicable, the number of events), effect estimates and confidence intervals. These may be tabulated or included on a forest plot. | Not applicable |
| Results of syntheses | 21 | Present summary effects for each meta-analysis undertaken, including confidence intervals and measures of statistical heterogeneity. State whether the analysis was pre-specified, and report the numbers of studies and participants and, where applicable, the number of events on which it is based. | 9; Table 2 |
|  |  | When exploring variation in effects due to patient or study characteristics, present summary interaction estimates for each characteristic examined, including confidence intervals and measures of statistical heterogeneity. State whether the analysis was pre-specified. State whether any interaction is consistent across trials. |  |
|  |  | Provide a description of the direction and size of effect in terms meaningful to those who would put findings into practice. |  |
| Risk of bias across studies | 22 | Present results of any assessment of risk of bias relating to the accumulated body of evidence, including any pertaining to the availability and representativeness of available studies, outcomes or other variables. | Not applicable |
| Additional analyses | 23 | Give results of any additional analyses (e.g. sensitivity analyses). If applicable, this should also include any analyses that incorporate aggregate data for studies that do not have IPD. If applicable, summarise the main meta-analysis results following the inclusion or exclusion of studies for which IPD were not available. | Figure 5 |
| **Discussion** | | | |
| Summary of evidence | 24 | Summarise the main findings, including the strength of evidence for each main outcome. | 11-12 |
| Strengths and limitations | 25 | Discuss any important strengths and limitations of the evidence including the benefits of access to IPD and any limitations arising from IPD that were not available. | 15-16 |
| Conclusions | 26 | Provide a general interpretation of the findings in the context of other evidence. | 12-15 |
| Implications | A4 | Consider relevance to key groups (such as policy makers, service providers and service users). Consider implications for future research. | 13-15 |
| **Funding** | | | |
| Funding | 27 | Describe sources of funding and other support (such as supply of IPD), and the role in the systematic review of those providing such support. | 19 |

**Appendix Supplement 2.** Literature search strategy

Relevant studies, published from March, 2011 (date of our last search for the previous review) to August, 2016 (date last searched), were identified through electronic searches not limited to the English language using MEDLINE, EMBASE, Web of Science, and Cochrane databases. Electronic searches were supplemented by scanning reference lists of articles identified for all relevant studies (including review articles), by hand searching of relevant journals and by correspondence with study investigators. The computer-based searches combined search terms related to hip replacement, infection, and revision with focus on one- and two stage surgeries without language restriction.

1 exp Prosthesis-Related Infections/ or prosthesis-related infection*.mp. or exp Sepsis/ (101971)

2 exp Infection/ or exp Wound Infection/ or exp Surgical Wound Infection/ or infection*.mp. (1603015)

3 wound infection.mp. or exp Wound Infection/ (44687)

4 arthroplasty.mp. or exp Arthroplasty, Replacement/ or exp Arthroplasty/ or exp Arthroplasty, Replacement, Hip/ (52003)

5 exp Arthroplasty, Replacement, Hip/ or exp Arthroplasty, Replacement/ or Replacement.mp. (204405)

6 exp Hip/ or exp Arthroplasty, Replacement, Hip/ or hip.mp. (107123)

7 exp Hip Prosthesis/ or exp Arthroplasty, Replacement, Hip/ or hip replacement.mp. (31655)

8 exp Hip Prosthesis/ or total hip.mp. or exp Arthroplasty, Replacement, Hip/ (34732)

9 hip arthroplasty.mp. (12045)

10 total hip replacement.mp. or exp Arthroplasty, Replacement, Hip/ (20706)

11 exp Arthroplasty, Replacement, Hip/ or total hip arthroplasty.mp. (20637)

12 exp Arthroplasty, Replacement, Hip/ or exp Hip Prosthesis/ or hip prosthes*.mp. (30397)

13 1-stage.mp. (1576)

14 2-stage.mp. (2828)

15 one stage.mp. (8705)

16 two stage.mp. (15977)

17 one-stage.mp. (8705)

18 two-stage.mp. (15977)

19 single stage.mp. (4349)

20 single-stage.mp. (4349)

21 prosthesis exchange.mp. (15)

22 direct exchange.mp. (153)

23 direct-exchange.mp. (153)

24 Arthroplasty, Replacement, Hip/ or revision arthroplasty.mp. (18017)

25 exp Arthroplasty, Replacement, Hip/ or staged revision.mp. (17490)

26 reoperation.mp. or exp Reoperation/ (77278)

27 reimplantation.mp. or exp Replantation/ (10449)

28 1 or 2 or 3 (1606087)

29 4 or 5 or 6 or 7 or 8 or 9 or 10 or 11 or 12 (295528)

30 13 or 14 or 15 or 16 or 17 or 18 or 19 or 20 or 21 or 22 or 23 or 24 or 25 or 26 or 27 (129343)

31 28 and 29 and 30 (5795)

32 limit 31 to yr="2011 -Current" (1602)

Each part was specifically translated for searching the other databases (EMBASE, Web of Science, and Cochrane databases)

**Appendix Supplement 3.** Study variables requested from investigators

| **Broad classification** | **Variables** |
| --- | --- |
| Socio-demographic characteristics | Country in which study was carried out, age, sex, body mass index or (weight and height), and smoking status |
| Past medical/surgical history | Previous hip surgery, other joint surgery, and co-morbidities (e.g., history of diabetes), Charnley classification, Charlson index, ASA grade |
| Infection characteristics before revision surgery | Duration between index implantation and occurrence/diagnosis of infection, duration between diagnosis of infection and revision surgery |
| Baseline laboratory data for infection | CRP, ESR, leucocytes, causative organism, neutrophil count, white cell count, synovial fluid white cell count, IL-6 |
| Characteristics of surgical revision | Date of revision, type of re-implantation, type of fixation (cemented or uncemented), use of spacer, type of spacer (static or articulating), use of antibiotics in cement or spacer, time interval between stages for two-stage procedure, diagnosis of re-infection, date of diagnosis of re-infection or date of last follow-up for participants without re-infection, time to re-infection or last date of follow-up (days or years), antibiotics used, and duration of antibiotics |
| Intervention | One-stage or two-stage revision |
| Outcomes | Re-infection (primary outcome); pain, function, mobility, re-revision surgery, or death as secondary outcomes |

ASA, American Society of Anaesthesiologists; CRP, C-reactive protein; ESR, erythrocyte sedimentation rate; IL-6, intereleukin-6

**Appendix Supplement 4.** Characteristics of studies contributing data to the pooled analysis

| **Lead Author, Publication Date** | **Location** | **Year of study** | **Median (IQR) age (years)** | **Median (IQR) follow-up (years)** | **No. of re-infections** | **No. of participants** |
| --- | --- | --- | --- | --- | --- | --- |
| **One-stage** |  |  |  |  |  |  |
| Hughes, 1979 | USA | 1971-1975 | 68 (58-71) | 3.8 (23.4-5.3) | 1 | 14 |
| Cherney, 1983 | USA | 1971-1978 | 70 (68-74) | 5.5 (4.0-6.6) | 0 | 4 |
| Goodman, 1988 | USA | 1971-1982 | 73 (66-80) | 3.5 (2.7-4.2) | 0 | 2 |
| Raut, 1996 | UK | 1979-1990 | 61 (57-75) | 8.2 (5.7-10.3) | 1 | 15 |
| Rudelli, 2008 | Brazil | 1989-2000 | 65 (51-71) | 14.0 (10.0-16.0) | 2 | 32 |
| Ure, 1998 | USA | 1979-1990 | 63 (56-68) | 10.1 (6.7-13.0) | 0 | 20 |
| Wu, 2003 | Taiwan | NS | 37 (27-54) | 3.2 (2.8-3.8) | 1 | 13 |
| Garcia, 2005 | Spain | NS | 76 (70-80) | 0.3 (0.2-0.3) | 0 | 14 |
| Yoo, 2009 | South Korea | 1991-2005 | 49 (38-65) | 7.0 (4.9-9.7) | 1 | 12 |
| Jenny, 2014 | France | 2007-2010 | 77 (63-84) | 3.0 (1.1-4.6) | 11 | 65 |
| Zeller, 2014 | France | 2002-2010 | 71 (60-78) | 3.2 (2.2-5.5) | 8 | 157 |
| Bori, 2014 | Spain | 1998-2007 | 75 (69-78) | 3.4 (2.4-5.0) | 1 | 24 |
| Board, 2016* | UK | 1980-2015 | 68 (61-75) | 4.7 (1.8-9.5) | 62 | 512 |
| **Two-stage** |  |  |  |  |  |  |
| Hughes, 1979 | USA | 1971-1975 | 60 (52-68) | 3.8 (2.8-4.1) | 4 | 12 |
| Cherney, 1983 | USA | 1971-1978 | 57 (49-64) | 4.0 (3.1-5.2) | 10 | 29 |
| Goodman, 1988 | USA | 1971-1982 | 68 (43-71) | 4.6 (3.2-6.5) | 1 | 7 |
| Wilson, 1989 | USA | NS | 54 (43-73) | 3.0 (3.0-4.0) | 0 | 19 |
| Antti-Poika, 1989 | Finland | 1976-1985 | 58 (51-68) | 4.9 (2.8-8.3) | 6 | 36 |
| Berry, 1991 | USA | NS | 54 (41-64) | 2.6 (2.5-4.1) | 3 | 18 |
| Alexeeff, 1996 | Canada | 1983-1993 | 70 (58-77) | 3.3 (2.3-5.6) | 0 | 11 |
| Fehring, 1999 | USA | NS | NS | 2.3 (2.0-3.9) | 2 | 25 |
| Wagner, 1995 | Germany | 1991-1993 | 71 (55-81) | 2.0 (2.0-2.0) | 9 | 34 |
| Weber, 2000 | Switzerland | 1990-1994 | 72 (67-73) | 4.2 (3.1-5.0) | 0 | 14 |
| Magnan, 2001 | Italy | 1996-1999 | 74 (66-76) | 2.0 (2.0-2.0) | 2 | 10 |
| Yamamoto, 2003 | Japan | 1998-2002 | 65 (62-70) | 3.8 (1.5-4.5) | 0 | 17 |
| Takahira, 2003 | Japan | 1996-2000 | 67 (65-71) | 3.7 (1.8-4.1) | 1 | 9 |
| Piriou, 2003 | France | 1987-1997 | 64 (51-70) | 3.0 (2.0-7.2) | 3 | 30 |
| Durbhakula, 2004 | USA | 1998-2001 | 70 (63-81) | 2.0 (2.0-2.0) | 0 | 20 |
| Evans, 2004 | USA | 1995-2002 | 69 (60-75) | 2.0 (2.0-2.0) | 11 | 46 |
| Sudo, 2008 | Japan | 1998-2000 | 65 (55-74) | 2.0 (2.0-2.0) | 0 | 7 |
| Cordero-Ampuero, 2009 | Spain | 1997-2007 | 69 (57-78) | 8.4 (5.0-9.9) | 0 | 19 |
| Whittaker, 2009 | UK | 1998-2003 | 68 (64-73) | 4.0 (3.0-5.1) | 7 | 44 |
| Dairaku, 2009 | Japan | 2002 | 64 (60-66) | 2.0 (2.0-2.0) | 1 | 10 |
| Darley, 2009 | UK | 2006-2007 | 63 (54-76) | 2.0 (2.0-2.0) | 1 | 19 |
| Estes, 2010 | USA | 2002 | 64 (59-72) | 5.2 (2.4-7.1) | 0 | 4 |
| Anagnostakos, 2010 | Germany | 1999-2008 | 69 (64-75) | 4.3 (2.0-4.9) | 1 | 13 |
| Takigami, 2010 | Japan | 1999-2006 | 66 (57-73) | 3.7 (2.4-5.7) | 0 | 8 |
| Cabo, 2011 | Spain | 2004-2009 | 68 (63-78) | 2.5 (2.0-6.3) | 6 | 19 |
| Sorli, 2012 | Spain | 2007-2008 | 74 (66-80) | 5.1 (2.4-7.2) | 6 | 14 |
| Huang, 2012 | USA | 2000-2007 | 63 (56-75) | 3.2 (2.0-5.5) | 13 | 48 |
| Schwarzkopf, 2014 | USA | 2001-2011 | 64 (54-77) | 1.4 (0.6-3.3) | 9 | 64 |
| Chen, 2015 | Taiwan | 2001-2010 | 58 (47-69) | 7.8 (4.9-10.3) | 10 | 150 |
| Board, 2016* | UK | 1983-2015 | 68 (60-74) | 3.5 (1.7-7.0) | 11 | 106 |
| Masri, 2016* | Canada | 1997-2001 | 66 (57-74) | 3.3 (2.0-4.7) | 17 | 110 |

*, unpublished data; NS, not stated.

**Appendix Supplement 5.** Classification of infecting organisms based on difficulty in treating

| **Difficult to treat**  Fungi  Small colony variants  Pseudomonas, quinolone-resistant pseudomonas  Enterococcus species  Antibiotic resistant subspecies of staphylococci  Vancomycin resistant enterococci/staphylococci  Chinolon resistant enterococci  Methicillin resistant *Staphylococcus aureus*  Rifampicin-resistant staphylococci  Extended-spectrum beta-lactamases  other multi-drug resistant micro-organisms |
| --- |
| **Not difficult to treat**  Others |

Adapted from Wimmer MD, Randau TM, Friedrich MJ, et al. Outcome Predictors in Prosthetic Joint Infections--Validation of a risk stratification score for Prosthetic Joint Infections in 120 cases. *Acta Orthop Belg.* 2016;82(1):143-148 and Zimmerli W, Trampuz A, Ochsner PE. Prosthetic-joint infections. *N Engl J Med.* 2004;351(16):1645-1654

**Appendix Supplement 6.** Main references of studies contributing data to the pooled analysis

1. Raut VV, Siney PD, Wroblewski BM. One-stage revision of total hip arthroplasty for deep infection. Long-term followup. *Clin Orthop.* 1995;321:202-207.

2. Rudelli S, Uip D, Honda E, Lima AL. One-stage revision of infected total hip arthroplasty with bone graft. *J Arthroplasty.* 2008;23(8):1165-1177.

3. Ure KJ, Amstutz HC, S N, Schmalzried TP. Direct-exchange arthroplasty for the treatment of infection after total hip replacement. An average ten-year follow-up. *J Bone Joint Surg Am.* 1998;80-A(7):961-968.

4. Garcia S, Soriano A, Esteban P, Almela M, Gallart X, Mensa J. Usefulness of adding antibiotic to cement in one stage exchange of chronic infection in total hip arthroplasty. *Medicina Clinica.* 2005;125(4):138-139.

5. Hughes PW, Salvati EA, Wilson PD, Blumenfeld EL. Treatment of Subacute Sepsis of the Hip by Antibiotics and Joint Replacement Criteria For Diagnosis With Evaluation of Twenty-Six Cases. *Clin Orthop.* 1979;141:143-157.

6. Wu CC, Chen WJ. One-stage revision surgery to treat hip infected nonunion after stabilization with a sliding compression screw. *Arch Orthop Trauma Surg.* 2003;123(8):383-387.

7. Yoo JJ, Kwon YS, Koo KH, Yoon KS, Kim YM, Kim HJ. One-stage cementless revision arthroplasty for infected hip replacements. *Int Orthop.* 2009;33(5):1195-1201.

8. Jenny JY, Lengert R, Diesinger Y, Gaudias J, Boeri C, Kempf JF. Routine one-stage exchange for chronic infection after total hip replacement. *Int Orthop.* 2014;38(12):2477-2481.

9. Zeller V, Lhotellier L, Marmor S, et al. One-stage exchange arthroplasty for chronic periprosthetic hip infection: results of a large prospective cohort study. *Journal of Bone & Joint Surgery - American Volume.* 2014;96(1):e1.

10. Bori G, Munoz-Mahamud E, Cune J, Gallart X, Fuster D, Soriano A. One-stage revision arthroplasty using cementless stem for infected hip arthroplasties. *Journal of Arthroplasty.* 2014;29(5):1076-1081.

11. Whittaker JP, Warren RE, Jones RS, Gregson PA. Is prolonged systemic antibiotic treatment essential in two-stage revision hip replacement for chronic Gram-positive infection (Journal of Bone and Joint Surgery - Series B (2009) 91-B, (44-51)). *Journal of Bone and Joint Surgery - Series B.* 2009;91 (5):700.

12. Cordero-Ampuero J, Esteban J, Garcia-Cimbrelo E. Oral antibiotics are effective for highly resistant hip arthroplasty infections. *Clin Orthop.* 2009;467(9):2335-2342.

13. Piriou P, de Loynes B, Garreau de Loubresse C, Judet T. Use of combined gallium-technetium scintigraphy to determine the interval before second-stage prosthetic reimplantation in hip arthroplasty infection: a consecutive series of 30 cases. *Rev Chir Orthop Reparatrice Appar Mot.* 2003;89(4):287-296.

14. Fehring TK, Calton TF, Griffin WL. Cementless fixation in 2-stage reimplantation for periprosthetic sepsis. *J Arthroplasty.* 1999;14(2):175-181.

15. Evans RP. Successful treatment of total hip and knee infection with articulating antibiotic components: a modified treatment method. *Clin Orthop.* 2004;427:37-46.

16. Wilson MG, Dorr LD. Reimplantation of infected total hip arthroplasties in the absence of antibiotic cement. *J Arthroplasty.* 1989;4(3):263-269.

17. Yamamoto K, Miyagawa N, Masaoka T, Katori Y, Shishido T, Imakiire A. Clinical effectiveness of antibiotic-impregnated cement spacers for the treatment of infected implants of the hip joint. *J Orthop Sci.* 2003;8(6):823-828.

18. Magnan B, Regis D, Biscaglia R, Bartolozzi P. Preformed acrylic bone cement spacer loaded with antibiotics: use of two-stage procedure in 10 patients because of infected hips after total replacement. *Acta Orthop Scand.* 2001;72(6):591-594.

19. Antti-Poika I, Santavirta S, Konttinen YT, Honkanen V. Outcome of the infected hip arthroplasty. A retrospective study of 36 patients. *Acta Orthop Scand.* 1989;60(6):670-675.

20. Weber E, Cometta A, Blanc CH, Leyvraz PF. Review of infected total arthroplasties of the hip and knee-apropos of 28 cases. *Swiss Surgery.* 2000;6(6):335-342.

21. Schwarzkopf R, Mikhael B, Wright E, Estok DM, 2nd, Katz JN. Treatment failure among infected periprosthetic total hip arthroplasty patients. *The open orthopaedics journal.* 2014;8:118-124.

22. Cabo J, Euba G, Saborido A, et al. Clinical outcome and microbiological findings using antibiotic-loaded spacers in two-stage revision of prosthetic joint infections. *J Infect.* 2011;63(1):23-31.

23. Cherney DL, Amstutz HC. Total hip replacement in the previously septic hip. *J Bone Joint Surg Am.* 1983;65-A(9):1256-1265.

24. Goodman SB, Schurman DJ. Outcome of infected total hip arthroplasty. An inclusive, consecutive series. *J Arthroplasty.* 1988;3(2):97-102.

25. Berry DJ, Chandler HP, Reilly DT. The use of bone allografts in two-stage reconstruction after failure of hip replacements due to infection. *J Bone Joint Surg Am.* 1991;73-A(10):1460-1468.

26. Alexeeff M, Mahomed N, Morsi E, Garbuz D, Gross A. Structural allograft in two-stage revisions for failed septic hip arthroplasty. *J Bone Joint Surg Am.* 1996;78-B(2):213-216.

27. Wagner M. Local antisepsis in revision surgery of infected total hip prostheses. *Orthopade.* 1995;24(4):319-325.

28. Takahira N, Itoman M, Higashi K, Uchiyama K, Miyabe M, Naruse K. Treatment outcome of two-stage revision total hip arthroplasty for infected hip arthroplasty using antibiotic-impregnated cement spacer. *J Orthop Sci.* 2003;8(1):26-31.

29. Durbhakula SM, Czajka J, Fuchs MD, Uhl RL. Spacer endoprosthesis for the treatment of infected total hip arthroplasty. *J Arthroplasty.* 2004;19(6):760-767.

30. Sudo A, Hasegawa M, Fukuda A, Uchida A. Treatment of infected hip arthroplasty with antibiotic-impregnated calcium hydroxyapatite. *J Arthroplasty.* 2008;23(1):145-150.

31. Dairaku K, Takagi M, Kawaji H, Sasaki K, Ishii M, Ogino T. Antibiotics-impregnated cement spacers in the first step of two-stage revision for infected totally replaced hip joints: report of ten trial cases. *J Orthop Sci.* 2009;14(6):704-710.

32. Darley E, Bannister G, Blom A, MacGowan AP, Jacobson S, Alfouzan W. Early intravenous to oral antibiotic switch therapy is effective in the treatment of infected total hip replacement. *Clin Microbiol Infect.* 2009;15(Suppl S4):S139.

33. Estes CS, Beauchamp CP, Clarke HD, Spangehl MJ. A two-stage retention debridement protocol for acute periprosthetic joint infections. *Clin Orthop.* 2010;468(8):2029-2038.

34. Anagnostakos K, Jung J, Kelm J, Schmitt E. Two-stage treatment protocol for isolated septic acetabular cup loosening. *Hip international : the journal of clinical and experimental research on hip pathology and therapy.* 2010;20(3):320-326.

35. Takigami I, Ito Y, Ishimaru D, et al. Two-stage revision surgery for hip prosthesis infection using antibiotic-loaded porous hydroxyapatite blocks. *Arch Orthop Trauma Surg.* 2010;130(10):1221-1226.

36. Sorli L, Puig L, Torres-Claramunt R, et al. The relationship between microbiology results in the second of a two-stage exchange procedure using cement spacers and the outcome after revision total joint replacement for infection: the use of sonication to aid bacteriological analysis. *J Bone Joint Surg Br.* 2012;94(2):249-253.

37. Huang R, Hu CC, Adeli B, Mortazavi J, Parvizi J. Culture-negative periprosthetic joint infection does not preclude infection control. *Clinical Orthopaedics & Related Research.* 2012;470(10):2717-2723.

38. Chen SY, Hu CC, Chen CC, Chang YH, Hsieh PH. Two-Stage Revision Arthroplasty for Periprosthetic Hip Infection: Mean Follow-Up of Ten Years. *Biomed Res Int.* 2015;2015:345475.
